# Low dose of apatinib in treating chemotherapy and EGFR-TKI refractory non-small cell lung cancer: A case report

**DOI:** 10.1097/MD.0000000000014328

**Published:** 2019-02-01

**Authors:** Jin Liu, Yulong Zheng, Nong Xu

**Affiliations:** Department of Medical Oncology, First Affiliated Hospital, School of Medicine, Zhejiang University, Hangzhou, China.

**Keywords:** anti-angiogenesis, apatinib, non-small cell lung cancer

## Abstract

**Rationale:**

Lung cancer is the leading cause of cancer-associated deaths all over the world. Although the prognosis of lung cancer has improved over the past decade due to progression in surgical techniques and systematic treatments, the patients with advanced disease still suffer poor survival. There are no standard treatment strategies for patients who have failed to respond to at least 2 lines of chemotherapy in non-small cell lung cancer (NSCLC). Apatinib, one of the latest small-molecule oral anti-angiogenesis targeted agents developed first in China, has shown remarkable anti-tumor efficacy in a variety of solid tumor types.

**Patient concerns:**

A 72-year-old woman underwent radical resection of the left lung cancer in July 2011, but was found a recurrence of cancer after 2 years.

**Diagnoses:**

The histopathological examination of the resected specimen identified the lesion as lung adenocarcinoma.

**Interventions:**

She received gemcitabine and carboplatin regimen as adjuvant chemotherapy for 4 cycles following the surgery in August 2011. After the tumor relapsed, she received multiple lines of chemotherapy including paclitaxel, cisplatin, docetaxel, and gemcitabine from July 2013, but still suffered progressive disease in February 2017. Then apatinib alone was used to defend against the tumor at a dose of 250 mg/d orally till December 2017.

**Outcomes:**

The efficacy was assessed as partial response 1 month later in March 2017. And the use of apatinib was continued till the patient died of tumor progression, achieving a progression-free survival for 10 months. During the treatment with apatinib, the patient experienced hypertension of grade 1, which was well-tolerated and manageable.

**Lessons:**

Apatinib might be efficient and well-tolerated for patients with advanced NSCLC who have failed to respond to multi-line treatments, even at a low dose.

## 1 Introduction

Lung cancer results in the largest number of cancer-related deaths worldwide, and more than 85% of the populations are diagnosed as non-small cell lung cancer (NSCLC). For lung cancer with all stages at diagnosis, the 5-year survival rate is only 16.8% and much lower for those with advanced disease, approximately 2%.^[1]^ NSCLC at early stage is primarily treated by surgical resection combined with adjuvant chemotherapy for selected patients, whereas advanced NSCLC remains an incurable disease, which should be managed comprehensively based on systemic therapy. As backbone of treatment in advanced NSCLC, platinum-based doublet chemotherapy does have clear clinical benefits, but seems to have reached the bottleneck in efficacy due to the limited benefits in overall survival (OS). Targeted therapies for patients with mutated EGFR and ALK have shown better results when compared with chemotherapy; however, most of them gain drug resistance inevitably and still have to undergo chemotherapy. Thus, making new strategies to treat patients with advanced NSCLC who suffered PD after 2 or more lines of chemotherapy is urgent.

Angiogenesis is a key process for cell growth. And vast data have shown that it plays a pivotal role in tumor growth, progression, local invasion, and distant metastasis.^[2]^ Based on this theory, anti-angiogenesis has been one of the most promising anti-cancer means. Apatinib, a small molecule oral anti-angiogenesis biologic agent targeting vascular endothelial growth factor receptor-2 (VEGFR-2), has been studied in multiple solid tumors and shown tremendous antitumor efficacy. Owing to favorable side effects profile and prolonged PFS and OS in advanced gastric cancer, apatinib has been approved to treat patients with advanced gastric cancer and adenocarcinoma in the gastroesophageal junction who failed to 2 or more lines of prior chemotherapy in China.^[3]^ Several studies focusing on efficacy and safety of apatinib in treating patients with breast cancer also obtained positive results.^[4,5]^ However, for lung cancer, such clinical trials and clinical practice are relatively rare. Herein, we report an old woman with lung adenocarcinoma who received apatinib as fourth-line treatment and got long PFS.

## 2 Case presentation

**Figure 1:**
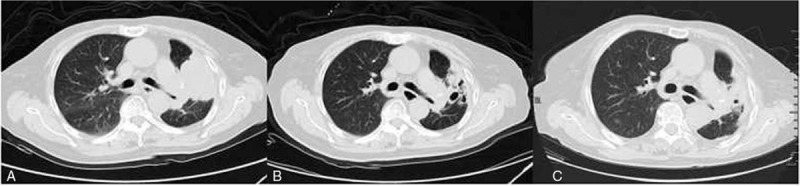
A: Metastatic lesions in the left lung and left pleural before apatinib usage on February 8, 2017 when failed to third-line chemotherapy; B: A month later on March 8, 2017, the metastasis in the left lung shrank by 25% compared with that a month ago, the efficacy was partial response. C: The tumor was evaluated as sable disease on August 21, 2017 after the use of apatinib for 6 months.

A 72-year-old female patient underwent radical resection of the left lung cancer in July 2011 because of a mass revealed by computed tomography (CT) of chest. Pathological examination confirmed the diagnosis of lung adenocarcinoma, with a stage of IIIA (pT1N2M0) based on the NCCN tumor-node-metastasis classification system. Then she proceeded with gemcitabine and carboplatin regimen as adjuvant chemotherapy for 4 cycles. During her routine review on July 19, 2013, thickened left pleura and small nodular lesions of both lungs were revealed by the chest CT, which were considered as tumor recurrence and intrapulmonary metastases. Subsequently, the patient was treated with paclitaxel and carboplatin as first-line chemotherapy for 4 cycles. She was also recommended to take icotinib orally at a dose of 125 mg 3× a day as maintenance therapy due to the active EGFR mutation (L858R in exon 21) found in November 2013. Unfortunately, in October 2014, the tumor was evaluated as PD again, which led to second-line chemotherapy involving docetaxel monotherapy for 4 cycles. Ten months later on August 27, 2015, the chest CT showed progressive tumor in the left lung and carcinoembryonic antigen (CEA) also increased; so, gemcitabine and cisplatinum were prescribed as third-line chemotherapy. However, the regimen was discontinued on the first day due to her severe nausea, vomiting, anorexia, and fatigue. Then the strategy switched to paclitaxel monotherapy for 4 cycles. At the same time, the genetic analysis using her peripheral blood sample displayed that T790 M was negative, so icotinib was continued as maintenance therapy. More than 1 year later on February 8, 2017, the patient was admitted to our department complaining of severe cough and white sputum with an Eastern Cooperative Oncology Group (ECOG) performance status (PS) of 2. The chest CT identified PD in left lung (Fig. 1A) and serum CEA level markedly increased to 715.3 ng/mL. No evidence of metastasis was observed in abdomen, brain, and bone with the CT examination or bone scan. After comprehensive assessment, apatinib alone was prescribed at a dose of 250 mg/d orally to defend against the tumor. Remarkable tumor regression (Fig. 1B) was observed, and CEA also sharply decreased to normal level 1 month later on March 8, 2017. Therefore, the regimen was continued and the chest CT was performed every 2 months to assess the tumor. On October 23, 2017, the chest CT indicated stable disease in the left lung (Fig. 1C). But 2 months later on December 15, 2017, the woman attended our hospital for chest pain with a bad ECOG PS of 3. The chest CT verified PD of left lung cancer and serious infection of both lungs. Although received anti-infective and supportive treatment, she died of respiratory failure 2 weeks later. Hypertension of grade 1 occurred in her whole course of apatinib treatment, which was tolerated and manageable with no dose adjustment or drug withdrawal. What deserves to be mentioned was that the patient had been taking icotinib as maintenance therapy between line-to-line chemotherapy until the administration of apatinib in her whole course of anti-tumor treatment.

## 3 Discussion

New blood vessel provides oxygen and nutrition for tumor cell growth, contributing to tumor survival, local invasion, and distant metastasis. Anti-angiogenesis therapy has been greatly investigated since the 1970s when Folkman first put forward that targeting the angiogenesis could interfere in tumorigenesis.^[6]^ Vascular endothelial growth factor (VEGF) protein members and their receptors may be the most crucial factors involved in angiogenesis in malignant tumors. VEGF protein family consists of VEGF A, B, C, D, F, placental growth factor (PIGF), and their receptors VEGFR-1, 2, 3, among which VEGF-A (or VEGF) and VEGFR-2 are widely accepted as mostly responsible for tumor development.^[7]^

Apatinib is an orally administered small molecule inhibitor of VEGFR-2, which binds to VEGFR-2 and inhibits VEGF binding and subsequent VEGFR-2 autophosphorylation, while VEGFR-2 undergoes autophosphorylation when stimulated by VEGF, inducing a signal transduction cascade that finally leads to vascular endothelium proliferation and survival.^[8]^ Apatinib can also reverse multidrug resistance through inhibiting transport function of multidrug resistance proteins and enhance the antitumor effect of epidermal growth factor receptor-tyrosine kinase inhibitors (EGFR-TKIs) after T790M.^[9,10]^

In terms of lung cancer, a retrospective study reported that NSCLC patients who received apatinib monotherapy as third-line treatment achieved an objective response rate (ORR) of 18.2%, a disease control rate (DCR) of 95.5%, and a median PFS of 6.7 months. The major adverse effects including hypertension, gastrointestinal reactions and hand-foot syndrome were controllable and tolerable.^[11]^ In another retrospective study, 25 patients with advanced NSCLC took apatinib alone at a dose of 500 mg/d as palliative therapy.^[12]^ The overall ORR and DCR were 8.0% and 68.0%, respectively. The overall median PFS was 5.17 months (95% CI: 0.76–9.57). For 13 patients in the second-line setting, the median PFS was 7.37 months (95% CI: 0.01–14.72), and for the 12 patients in the third-line or beyond therapy, which was 5.17 months (95% CI: 1.78–8.55). All patients had good tolerance to apatinib and no grade 3/4 adverse events occurred. Zeng found that patients with lung adenocarcinoma who received apatinib monotherapy at a small dose (250–500 mg/d) as second-line treatment finally obtained an ORR of 18.75%, a DCR of 68.75%, and a median PFS of 4.4 months. Main toxicities such as hypertension, hand-foot syndrome, proteinuria, and thrombocytopenia were also tolerable and manageable.^[13]^

For our patient, apatinib was prescribed at a low dose of 250 mg/d, considering the female's failure to multi-line chemotherapy and ECOG PS of 2. Generally, apatinib at an initial dose of 750 mg/d shows clinical benefits in advanced NSCLC, and a dose of 500 mg/d is more commonly adopted in previous trials in case of unbearable adverse events.^[14,15]^ Dose modification to 250 mg/d in advanced NSCLC was also reported in literature before and the patient even achieved PR just like our case.^[16]^ Our patient finally achieved a PFS of 10 months, which was encouraging, as the median PFS of apatinib in treating advanced NSCLC was approximately 5 months according to previous reports.^[11–13]^ The most common adverse effects of apatinib are hypertension, hand-foot syndrome, and proteinuria. The patient experienced only hypertension of grade 1 in the course of using aptinib, which might be attributed to the small dose.

Our patient used another targeted drug icotinib as maintenance therapy in her whole course of antitumor treatment. It has been thought that apatinib could enhance efficacy of EGFR-TKIs in advanced NSCLC, however, whether prior use of EGFR-TKIs makes positive influence on efficacy of apatinib and contributes to our patient's long PFS are still unclear and need to be confirmed in the future.

Therefore, our case suggests that apatinib might be efficient and well-tolerated for advanced NSCLC patients who failed to respond to multi-line chemotherapy, even at a low dose. However, this is only a case report and more clinical trials should be launched to confirm.

## Acknowledgments

The authors acknowledge the support of the Department of Radiology, the First Affiliated Hospital, Zhejiang University School of Medicine.

## Author contributions

**Conceptualization:** Jin Liu, Nong Xu.

**Data curation:** Yulong Zheng.

**Resources:** Jin Liu.

**Supervision:** Yulong Zheng.

**Visualization:** Nong Xu.

**Writing – original draft:** Jin Liu.

**Writing – review & editing:** Nong Xu.
